# Requirements for a successful implementation of liver transplantation as a treatment option for non-resectable colorectal liver metastases into clinical routine – a narrative review

**DOI:** 10.1007/s00423-026-04006-5

**Published:** 2026-03-20

**Authors:** Kilian Alexander Walter, Simon Moosburner, Tabea Kreutz, Joseph MGV Gassner, Robert Öllinger, Felix Krenzien, Dominik Paul Modest, Markus Guba, Andreas Pascher, Johann Pratschke, Nathanael Raschzok

**Affiliations:** 1 Department of Surgery, Campus Charité Mitte | Campus Virchow Klinikum, Charité, Universitätsmedizin Berlin, corporate member of Freie Universität Berlin and Humboldt-Universität zu Berlin, Augustenburger Platz 1, Berlin, 13353 Germany; 2 BIH Biomedical Innovation Academy, BIH Charité Clinician Scientist Program, Berlin Institute of Health at Charité, Universitätsmedizin Berlin, Berlin, Germany; 3 Department of Hematology, Oncology, and Cancer Immunology (CVK/CCM), Charité-Universitätsmedizin Berlin, corporate member of Freie Universität Berlin and Humboldt-Universität zu Berlin, Berlin, Germany; 4 Charité Comprehensive Cancer Center (CCCC), Charité– Universitätsmedizin Berlin, corporate member of Freie Universität Berlin and Humboldt-Universität zu Berlin, Berlin, Germany; 5 Department of General, Visceral and Transplantation Surgery, LMU University Hospital, LMU Munich, Munich, Germany; 6 Department of General, Visceral and Transplant Surgery, University Hospital Muenster, Muenster, Germany

**Keywords:** Colorectal cancer, Colorectal liver metastases, Liver transplantation, Living donor liver transplantation, Deceased donor liver transplantation

## Abstract

**Purpose:**

Colorectal cancer remains a major challenge for the global health care system. Many patients with colorectal cancer develop liver metastases (CRLM), and a certain proportion of these patients are neither eligible for surgical nor interventional treatment. Liver transplantation (LT) could be a curative treatment option for these patients.

**Methods:**

This narrative review was based on a targeted literature search of the National Library of medicine (PubMED) to identify current publications investigating LT as a treatment option for patients with CRLM without local treatment options. The search term “colorectal AND liver transplantation” was used, and only original articles of the last ten years were included. Additionally, the ClinicalTrials.gov registry was screened for ongoing clinical trials in this field.

**Results:**

A total of 6 studies were identified, along with 21 active clinical trials. LT was associated with higher survival rates compared to standard of care, e.g., 73.3% 5-year survival after LT plus chemotherapy vs. 9.3% after chemotherapy alone in the randomized TransMET trial. Candidates eligible for LT were most often identified based on stable, liver-limited disease under active chemotherapy, in addition to further strict selection criteria such as favorable molecular pathology and metabolic tumor volume. Patients were accordingly prioritized on the waiting list. Long-term follow-up revealed substantial recurrence rates across the studies, most occurring in the lungs. Despite these recurrences, overall survival remained superior to standard of care.

**Conclusion:**

The reviewed evidence suggests that LT should be considered as part of a multimodal treatment algorithm for highly selected patients with non-resectable CRLM. This paradigm shift has already translated into several ongoing trials on this subject. Policy adjustments in organ allocation and the development of structured programs are necessary to establish LT for CRLM patients in clinical routine.

## Introduction

Colorectal cancer (CRC) is one of the most prevalent malignancies worldwide, ranking as the third most common diagnosed cancer and the second leading cause of cancer‐related mortality [1, 2]. According to the Global Cancer Observatory, the worldwide incidence of CRC in 2022 was as high as 1,926,425 cases per year, surpassed only by malignancies of the respiratory tract and breast cancer. Current statistical models suggest that these numbers will further increase, with an estimated incidence of approximately 3.6 million cases annually in 2050 [3]. While advances in screening, early detection, and treatment options have improved overall survival, approximately 50% of all patients eventually develop metastatic disease, which remains a significant barrier to long-term survival [4, 5].

The liver is one of the most common sites for distant metastases from CRC due to its direct anatomical link via the portal blood flow, which exposes it to circulating tumor cells [6]. Currently, the management of colorectal liver metastases (CRLM) is mainly determined by local treatment of these lesions, either by surgical resection or interventional treatment such as ablation, combined with systemic therapy. Eligibility for local treatment is determined by multiple factors, such as tumor burden, anatomical distribution, extrahepatic disease, and the functional reserve of the future liver remnant. In addition, local liver-directed therapies such as radiofrequency ablation, transarterial chemoembolization (TACE) and stereotactic body radiotherapy (SBRT) are utilized to downstage tumors or as a definitive treatment options [7, 8] all being part of well-established treatment algorithms such as the ESMO clinical guidelines [9]. Non-resectability, or the inability to receive local treatment, is generally defined by extensive bilateral liver involvement, an insufficient liver remnant after resection, or the presence of extrahepatic disease that limits the effectiveness of a curative surgical approach [10]. Patients with CRLM without local treatment options face a poor prognosis, with median survival typically ranging between 6 and 12 months and with 5-year overall survival rates remaining well below 50% [11, 12]. When local therapy is not a viable option, systemic chemotherapy alone, with or without targeted therapies like anti-VEGF agents (e.g., bevacizumab) or anti-EGFR agents, represents the usual course of treatment [13]. However, long-term survival is rarely achieved in these patients.

Liver transplantation (LT) has long been established as a curative option for patients with end-stage liver disease and certain malignancies such as hepatocellular carcinoma (HCC) [14]. In these patients, replacing the diseased liver can effectively achieve complete removal of the malignancy while providing a graft with robust functional reserve. Historically though, LT was not considered for CRLM because of concerns over high recurrence rates as well as reported 1-and 5-year survival rate lower than the generally accepted threshold of 50% 5-year survival rate for transplant patients. Although these early findings seem disillusioning, this paradigm is being challenged by recent studies [15, 16].

Emerging evidence suggests that LT may offer a significant survival benefit for highly selected patients with non-resectable, liver-limited colorectal metastases compared to chemotherapy alone. Prospective studies have reported 5-year overall survival rates exceeding 50% after LT, a marked improvement over conventional systemic therapy [17]. In addition, there is early evidence suggesting that the therapeutic regimens administered posttransplant may further contribute to improved disease control [18].

This narrative review will explore the current state of knowledge regarding LT as an innovative therapeutic option for patients with CRLM without local treatment options. By examining recent advances, patient selection strategies, and long-term outcomes, we aim to evaluate the potential of LT in improving survival of CRLM patients and to build a framework for development of structured clinical transplant programs for this patient population.

## Materials and methods

### Search strategy, quality assessment

This narrative review is based on a targeted literature search of the National Library of Medicine (PubMed) to identify publications addressing liver transplantation (LT) as a treatment option for patients with colorectal liver metastases (CRLM). The search was last updated on March 16, 2025. The following search term was used:

Colorectal AND liver transplantation.

**Fig. 1:**
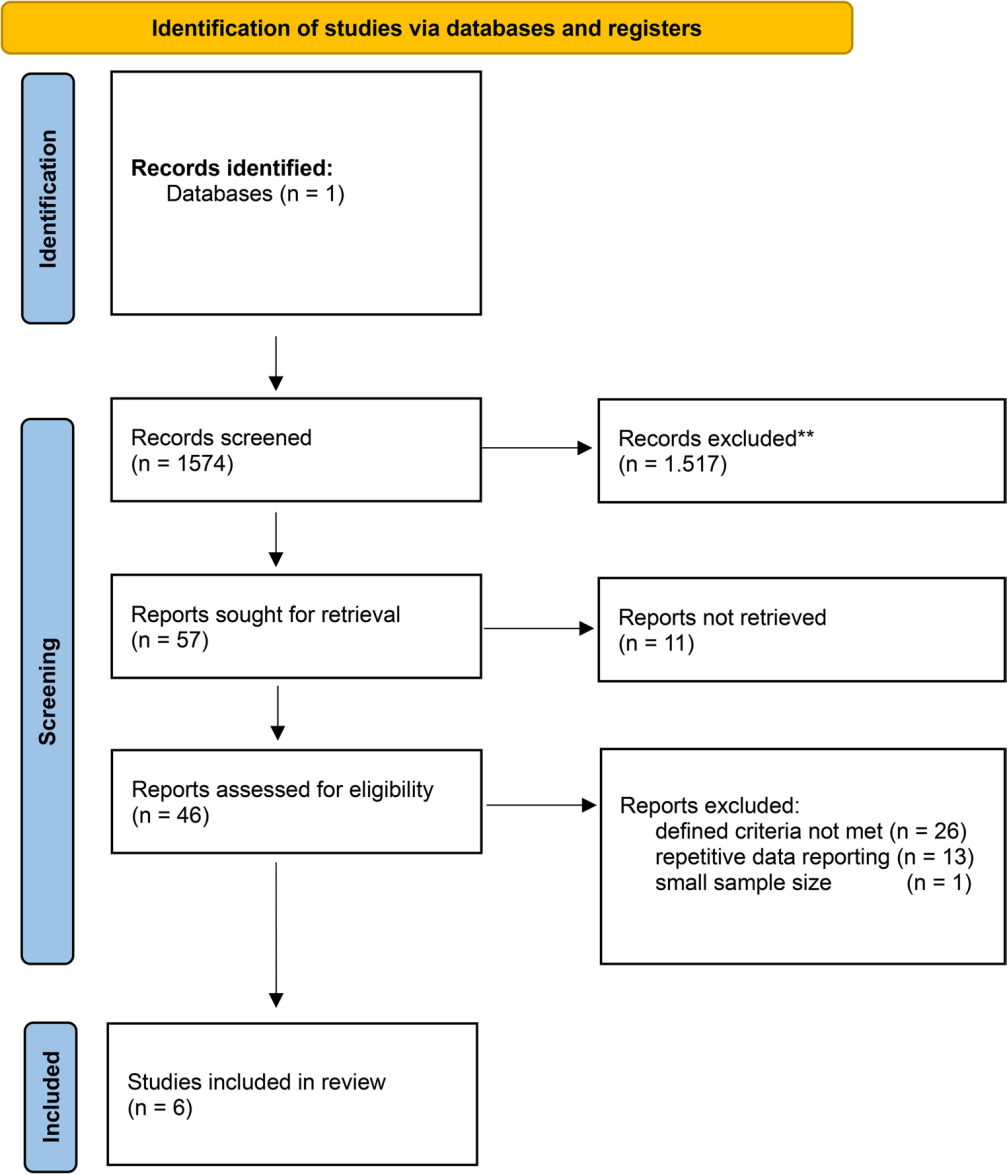
Flowchart of paper identification

Included in this review were original publications investigating LT as a treatment option for patients with CRLM. Excluded were reviews, metaanalyses, comments, editorials, letters, case reports, conference abstracts, and video articles. Articles for which the full text was not available in German or English were also excluded. To ensure an accurate representation of current trends, the scope of the search was limited to publications from the past ten years that reported a minimum of five cases. Titles and abstracts were screened for relevance, and the full texts of potentially eligible articles were reviewed for inclusion. Subsequently, during the second phase, the full-text articles of the remaining studies were screened and evaluated. Two independent evaluators (KAW and TK) were responsible for the review process of the remaining studies (Fig. 1).

In addition, the ClinicalTrials.gov registry of the U.S. National Library of Congress were searched on October 31st, 2025, using the following search terms: “liver transplantation” and “colorectal cancer”. Inclusion criteria for clinical trials involved any kind of trials on LT for patients with CRLM with the following recruitment statuses: “recruiting”, “active, not recruiting”, “not yet recruiting” or “enrolling by invitation”.

**Fig. 2:**
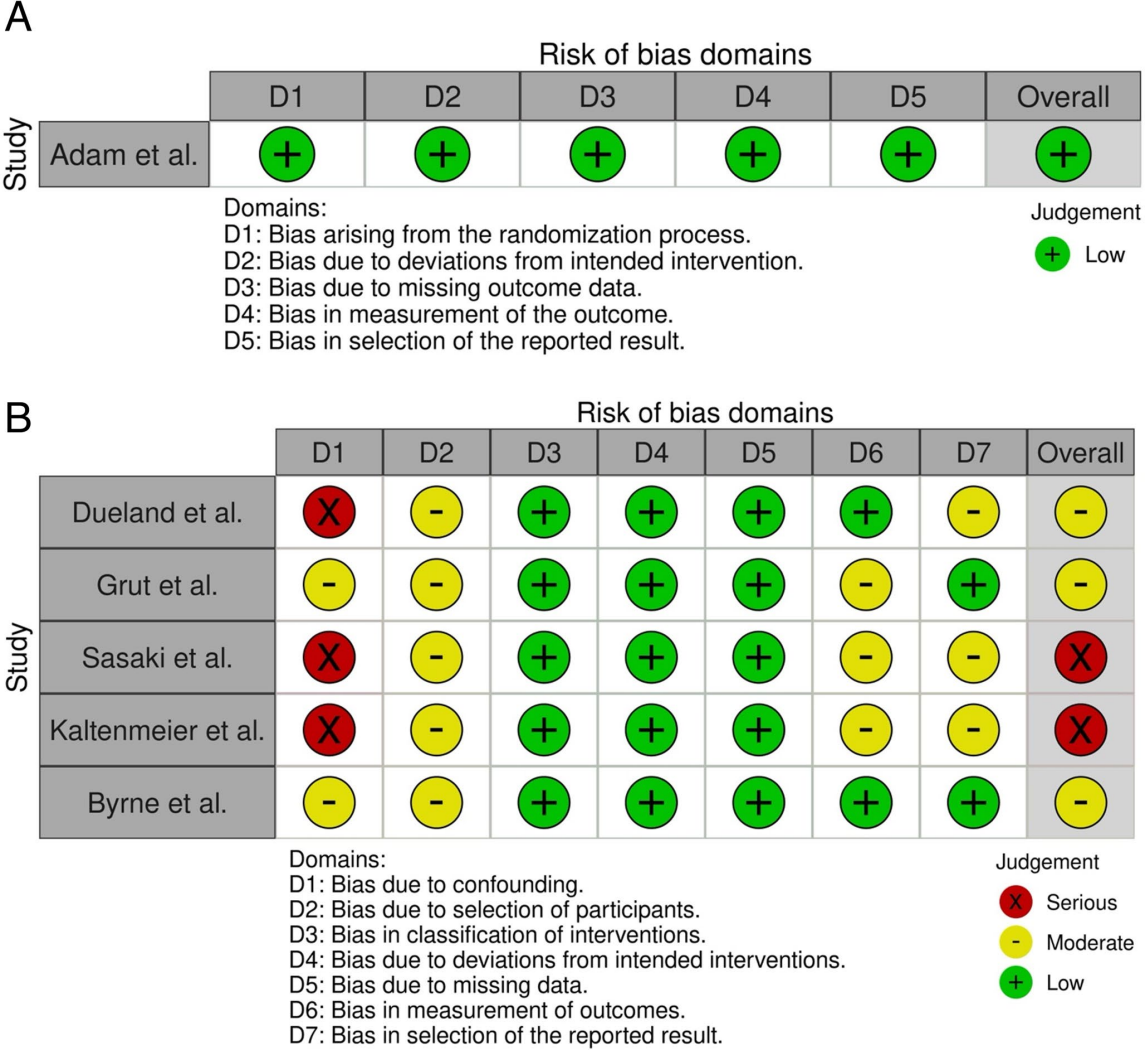
Risk of bias tools

Although this was a narrative review, the authors still opted to assess the quality of the included publications using the revised Cochrane risk-of-bias tool for randomized trials (RoB 2) [19]. In cases where it was not applicable, the Risk of Bias in Non-randomized Studies - of Interventions tool (ROBINS-I) was used [20, 21] (Fig. 2).

## Results

### Search results

Following the search strategy outlined above, an initial search produced 1574 results. Based on title and abstract screening, 1517 studies were excluded, including duplicates. In total, 57 studies were identified after abstract screening. For 11 of these studies, the full text was not retrieved. In two cases, no full text was found online even after extensive search. The remaining 9 studies were accessible only through paid access requiring substantial royalty fees. Due to financial limitations, these papers were not accessed. For the remaining 46 studies, the full text was retrieved and screened. Following this step, another 26 studies were excluded (i.e. for repetitive data reporting). The remaining 20 studies were evaluated by two independent authors regarding their value for the scope of this review. Where no consensus could be reached, a third author (SM) was consulted as an expert, and consensus was achieved through discussion. Ultimately, six publications (Table 1), were chosen for in-depth data extraction [22–27].

**Table 1:** Overview of included studies

Author, year	PMID, Ref.	type	study period	mode of LT	*N*	No. of patients receiving LT	median MELD Score	Chemotherapy regimen	1-/3-/5-year survival	median survival	1-/3-/5-year disease-free survival	median survival after relapse	median time to relapse	median recurrence	median disease-free survival
Matthew M. Byrne, 2024	39,332,681 [22]	Prospective Single-Center Cohort Study	2019–2024	LDLT	206	23	/	not reported	100%/91%/-	/	100%/40%/-	/	/	/	/
René Adam, 2024	39,306,468 [23]	Randomized Controlled Trial (RCT)	2016–2021	DDLT	94	36	/	Doublet (68%), Triplet (32%), targeted therapy	-/-/−56.6% (intention-to-treat-analysis) 73.2% (per-protocol-analysis) 12% Control group	/	-/32.9%/19.9%	/	17.4 mo.	72%	/
Christof Kaltenmeier, 2024	37,805,187 [24]	Retrospective Single-Center Cohort Study	2019–2022	LDLT	10	10	11	FOLFOX, FOLFIRI, FOLFOXIRI, targeted therapy	-/-/-	3 years	-/70%/-	/	/	/	2.2 years
Svein Dueland, 2023	37,494,056 [25]	Prospective Non-randomized Controlled Cohort	2006–2020	DDLT	61	61	/	not reported	-/-/50.4%	60.3 months	-/-/18.3%	37.1 months	9.0 mo.	/	11.8 mo.
Kazunari Sasaki, 2023	36,719,568 [26]	Retrospective Registry-Based Cohort Study	2017–2022	LDLT (56.5%) DDLT (43.5%)	64	46	8 (LDLT) 12 (DDLT)​	not reported	89%/60.4%/-	/	75.1%/53.7%/-	/	/	/	/
Harald Grut, 2022	36,241,941 [27]	Retrospective Cohort Analysis	2006–2018	DDLT	40	40 26 MTV low 14 MTV high	/	not reported	-/ 76% MTV low, 21% MTV high/50% MTV low, 7% MTV high	53% MTV low 36% MTV high	31% MTV low, 0% MTV high 21% MTV low, 0% MTV high 57% MTV low, 14% MTV high	/	/	16 mo. MTV low 4 mo. MTV high	

Legend: *LDLT* Living donor liver transplantation, *DDLT* Deceased donor liver transplantation, *LT* Liver transplantation, *MTV 2* Metabolic tumor volume, *MELD* Model for End-Stage liver disease

### Quality of studies

Of the studies included, only the TransMet trial published by Adam et. al. was a randomized controlled trial (RCT). Using the RoB2 tool, an overall low risk of bias was found, owing to it being a multicenter study with clear randomization, comprehensive follow-up, and proper intention-to-treat analysis. Conversely, some of the selected studies had inherent methodological constraints. The large pooled prospective cohort conducted by Dueland et al. in 2023 [25] demonstrated a comprehensive follow-up but lacked adequate control groups. Similarly, the SECA study assessing metabolic tumor volume [27] was limited by small sample size. The American cohort analyses from Pittsburgh by Kaltenmeier et al. [24] included only limited numbers of patients being transplanted. Finally, a retrospective U.S. national database analysis by Sasaki et al. [26]was constrained by its observational retrospective design.

### Patient demographics and clinical status

Sample size varied, ranging from smaller cohorts of 10 patients in specialized single-center studies [24] to larger, multicenter trials encompassing as many as 94 patients [23]. In the case of Bryne et al., a total of 206 patients were screened for eligibility, with 23 patients receiving a transplant. Among eligible candidates aged between 18 and 65 years, the median patient age ranged from 43 years [22] up to 58 years [24]. Across the studies investigated, patients had an overall good clinical status, such as an ECOG score of 0–1 as well as good liver function measured by median MELD scores consistently below 12 [26].

#### Primary tumor, criteria for non-resectability, and waiting time to transplantation

As a prerequisite for inclusion in the respective trials, all studies required patients to be diagnosed with histologically proven colon or rectal carcinoma. Resection of the primary colorectal tumor was required in all studies prior to transplantation. The interval between primary tumor resection and subsequent LT varied, typically reflecting protocol-specific inclusion criteria and treatment strategies. Median intervals reported were approximately 17 months (range 2–111 months) in the Oslo SECA studies by Grut et al. in 2022 [27] and approximately 16.9 months (range 2.3–173.8 months) in the larger Oslo cohort by Dueland et al. in 2023 [25]. In the North American studies by Kaltenmeier et al. [24] and Bryne et. al. [22], intervals tended to be longer, generally around 2–3 years. Specifically, Kaltenmeier et al. [24] reported a median interval of approximately 34 months (range 13.2–112.8 months), while Bryne et al. required at least 6–12 months, along with at least 12 months since CRLM diagnosis and sustained disease control. The TransMet trial by Adam et al. [23] also reported varied intervals, typically spanning from several months up to more than two years, with at least two months of mandatory postoperative chemotherapy of the primary was resected after inclusion to the trial. Overall, across centers, patients typically waited approximately 1.5–3 years from resection of the colorectal primary to LT.

As all studies explored LT as an option in patients with CRLM without local treatment options, i.e. surgery or ablation, a great emphasis was placed on establishing non-resectability. In clinical practice, non-resectability is defined as the inability to safely remove all tumors while leaving an adequate future liver remnant. Here, an R0 resection becomes impossible due to the risk of being left with too little residual liver, typically <20–30% of liver volume in normal livers [28]. Patients with multifocal, bilobar metastases often fall into this category. Tumor invasion of major inflow or outflow vessels is also deemed non-resectable. Regarding the studies investigated, non-resectability was usually defined by means of an expert panel decision, consisting of surgeons, oncologists, and radiologists.

### Transplantation modalities: living donor vs. deceased donor

European centers often utilized grafts from deceased donors (DDLT) [23, 25, 27]. In contrast, U.S. centers favored living-donor liver transplantation (LDLT), accounting for approximately 60% of transplantations in U.S. registry analyses [26] and 100% in dedicated LDLT cohorts from Pittsburgh and Rochester [22, 24].

#### Molecular, pathological, and clinical inclusion and exclusion criteria

As described above, patients were required to have undergone complete resection of the primary tumor according to established standards in oncologic surgery [23, 25]. The presence of CRLM at time of listing for LT was mandatory. Across all trials, patients were required to demonstrate an adequate response or at least stable disease after systemic chemotherapy, i.e. at least a 30% response by RECIST criteria [27].

Inclusion criteria also considered the tumor biology, excluding colorectal primary with detected BRAF V600E mutation or MSI-H/dMMR status, and set thresholds for carcinoembryonic antigen (CEA) levels or requiring a significant reduction from previous peaks [24, 27]. Accepted manuscriptMoreover, patients with known extrahepatic disease were excluded, as well as patients with progressive or chemotherapy-resistant tumors [23, 25]. High pre-transplant tumor burden, uncontrolled rising CEA levels, and aggressive mutations as mentioned above were also exclusionary. Additionally, patients with poor performance status, significant unintended weight loss (>10%), morbid obesity, or serious comorbidities contraindicating major surgery or immunosuppressive therapy were excluded [22, 27]. Further exclusion criteria were active or unresected primary colorectal tumors, concurrent or recent other malignancies, active viral infections (e.g., HIV, hepatitis B or C and pregnancy.

### Pretransplant chemotherapy and bridging therapies

Systemic chemotherapy prior to LT was universally applied across studies. Duration of chemotherapy typically ranged from 3 to 6 months, often employing combination regimens (FOLFOXIRI or FOLFIRI with biologic agents) to achieve maximum disease control [23, 24]. Some studies utilized additional bridging treatments such as hepatic artery infusion chemotherapy (HAI) and radiofrequency ablation (RFA), with up to 50% of patients receiving these additional therapies in some cohorts such as those by Kaltenmeier et al. [24] from 2024. The use of postoperative adjuvant chemotherapy varied between studies. Notably, in the TransMet study, 68% of the transplanted patients received additional chemotherapy after transplantation, with 15 (58%) of those patients treated with more than six cycles [23].

### Overall survival 

Across all studies investigated, LT was associated with higher survival rates compared to standard treatment, i.e. systemic chemotherapy alone. One-year overall survival (OS) in patients receiving LT was superior to those receiving standard of care, ranging from 89% in the multicenter U.S. registry study by Sasaki et al. [26] to a 100% in very selected LDLT cohorts [22]. Three-year OS varied considerably, ranging from as low as 21 % in patients with high metabolic tumor volume [27] to 91% in the LDLT Rochester cohort by Byrne et al. [22]. The TransMet trial by Adam et al. [23] confirmed this trend, reporting a 5-year OS of 56.6% (95% CI 43.2–74.1.2.1) among patients receiving transplantation and chemotherapy versus a 12.6% (5.2–30.1.2.1) with chemotherapy alone (HR 0.37; p=0.0003) in intention-to-treat and 73.3% (95% CI 59.6–90.0.6.0) vs. 9.3% (3.2–26.8.2.8) in per-protocol analyses.

### Recurrence rates, disease free survival, survival after relapse

Recurrence-free survival (RFS) rates varied between studies. At one-year posttransplantation, RFS rates had a large range; from around 50% in larger DDLT cohorts [25] to as high as 100% in highly selective patients undergoing LDLT [22]. However, longer-term follow-up revealed substantial recurrence rates: by five years post-transplantation, recurrence-free survival dropped to as low as 18.3% in some studies [25]. Recurrence typically occurred within the first year after transplantation, commonly between 8 to 12 months posttransplantation [25, 27]. Even though these findings seemed challenging, the investigated studies studies reported an overall favorable survival following recurrence, particularly when aggressive metastasis-directed therapies were employed. For example, the trial run by Dueland et al from 2023 reported a median survival after recurrence of approximately three years (37.1 months), with over 30% of patients still alive five years post-relapse. Similarly, in LDLT cohorts from Rochester and Pittsburgh, aggressive management of isolated metastases significantly contributed to overall survival despite recurrence rates of around 30%–60% within the first three years [22, 24].

### Overall mortality

Overall mortality following transplantation differed among studies. At five years, mortality varied from approximately 27% in LDLT studies such as Adam et al. [23] and Byrne et al. [22] to approximately 50% in broader DDLT cohorts such as Dueland et al. [25]. Notably, transplantation-related mortality was minimal, with most deaths attributable to disease progression rather than complications from the transplant procedure itself.

#### Metabolic tumor volume

Two out of the six selected studies considered metabolic tumor volume (MTV) [25, 27] as a prognostic marker for the success of LT in CRLM patients. In the study published by Grut et al. [27] preoperative 18F-FDG PET/CT from 40 patients with liver metastasis were evaluated. Study participants were divided into two groups, patients with less than 70 cm3 were considered as the low MTV group, with the other patients forming the high MTV group. When comparing both groups, patients with low MTV had significantly longer overall survival (76% vs. 21% 5-year OS, p < 0.001) compared to individuals with high values. Patients with high MTV had also a higher number of liver metastases as well as a larger size of the largest liver metastasis. An equally stark difference was reported by Dueland et. al in 2023 [25], with low MTV patients displaying 5-year OS-rates of 66.7% compared with patients with high MTV values who had a 5-year OS of 23.3% (p < 0.001).

### Currently active clinical trials

**Fig. 3:**
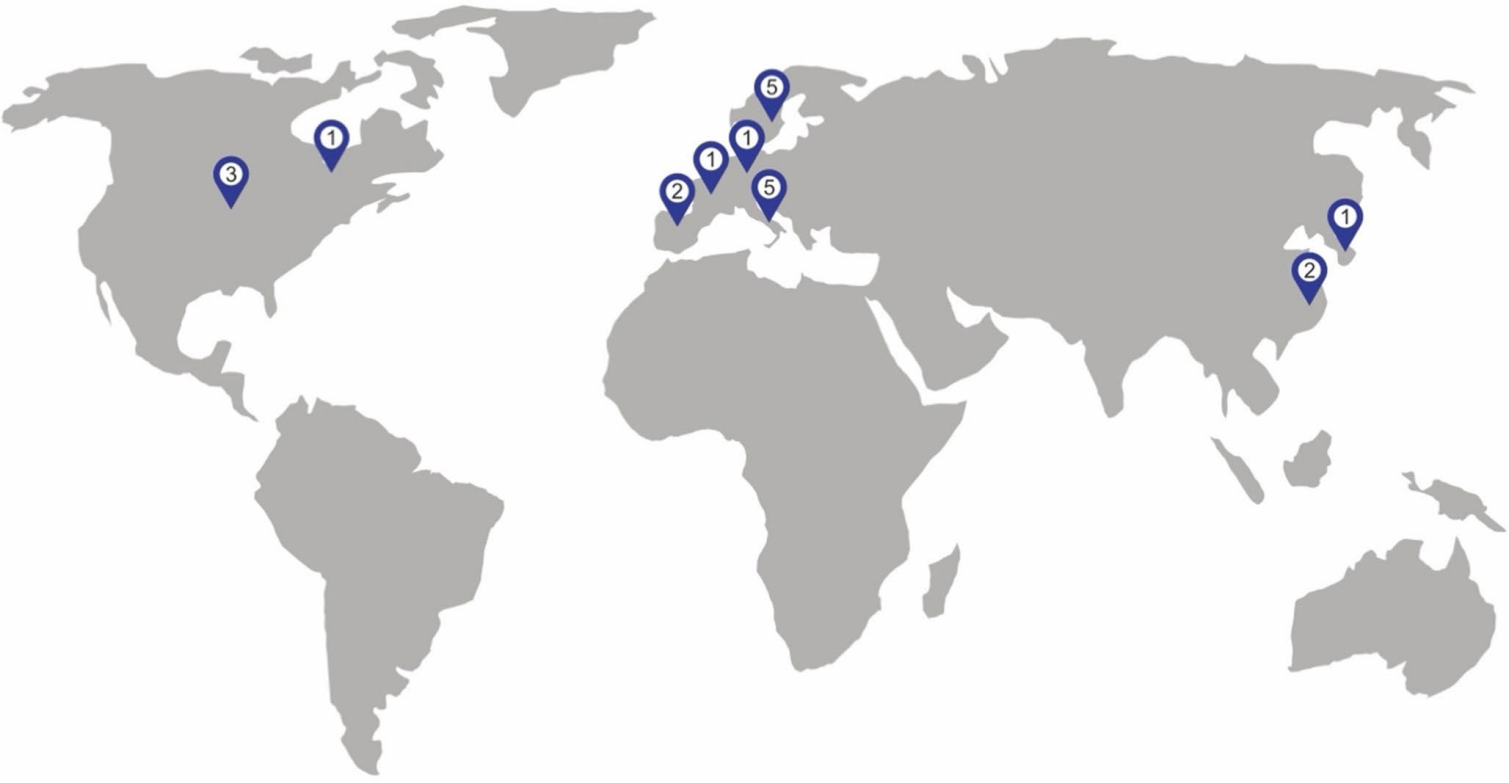
Locations of ongoing clinical trials

As of October 31 st, 2025, a total of 21 active clinical studies listed in the ClinicalTrials.gov registry investigating LT for patients with CRLM not amenable to local treatment were identified (Table 2, Fig. 3). Among these, the TRANSMET trial was of particular interest, although it is still active, recruitment was closed on July 5, 2021. Most ongoing studies require a waiting period of more than three months under chemotherapy. In a study conducted at the University of Toronto, only 7 of 85 enrolled patients were transplanted, because only these showed no hepatic progression over more than six months while on active chemotherapy. Among the non-transplanted patients in this cohort, 22 were subsequently able to undergo liver resection (trial-no: NCT02864485).

**Table 2:** Overview of screened clinical trials

NCT Number	Study Title	Acronym	Start Date	Locations
NCT00294827	Liver Transplantation and Metastatic Colo-rectal Cancer.	/	2006-02	Oslo, Norway
NCT04870879	Colorectal Metastasis and Liver Transplantation With Organs From Deceased Donors	MELODIC	2020-10-01	Padua, Italy
NCT04161092	The Swedish Study of Liver Transplantation for Non-resectable Colorectal Cancer Metastases^	SOULMATE	2020-12-01	Gothenburg & Stockholm, Sweden
NCT01479608	Liver Transplantation and Colorectal Cancer	/	2012-04-11	Oslo, Norway
NCT02215889	Partial Liver Segment 2/3 Transplantation Study	/	2014-06	Oslo, Norway
NCT05186116	LDLT in Non Resectable Colo-rectal Cancer Liver Metastasis	LIVERMORE	2022-01-01	Modena, Italy
NCT03494946	Liver Transplantation Compared to Chemotherapy in Patients With Colorectal Cancer	SECAIII	2016-12-05	Oslo, Norway
NCT04616495	Liver Transplantation in Patients With Unresectable Colorectal Liver Metastases	TRASMETIR	2021-09-01	Valencia, Spain
NCT05248581	Living Donor Liver Transplant for Unresectable Colorectal Liver Metastases	/	2019-08-16	Rochester, United States
NCT05185245	Liver Transplantation for Non-Resectable Colorectal Liver Metastasis	/	2021-04-01	Bologna, Italy
NCT03803436	Improving Outcome of Selected Patients With Non-resectable Hepatic Metastases From Colo-rectal Cancer With Liver Transplantation	COLT	2019-01-02	Ancona, Bergamo, Genua, Mailand, Pisa, Turin, Udine, Verona, Palermo, Italy
NCT02864485	Living Donor Liver Transplantation for Unresectable Colorectal Cancer Liver Metastases	/	2016-08-03	Toronto, Canada
NCT05750329	Liver Transplantation With Two-stage Liver Resection in Unresectable Liver Cancer, Metastases or End-stage Liver Disease	LTLR-LC	2023-08	Shanghai, China
NCT06698146	Colorectal Metastasis to Liver Extraction With Auxiliary Transplant and Delayed Resection	CLEAR	2025-06-01	Chicago, United States
NCT04874259	LT for Unresectable Colorectal Liver Metastasis	/	2022-08-22	Seoul, South Korea
NCT02597348	Liver Transplantation in Patients With Unresectable Colorectal Liver Metastases Treated by Chemotherapy	TRANSMET	2016-02	Villejuif, France
NCT05398380	Liver Transplantation for Non-resectable Colorectal Liver Metastases: Translational Research	METLIVER	2022-01-01	Barcelona, Spain
NCT04865471	Resection And Partial Liver Segmental Transplantation With Delayed Total Hepatectomy	RAPID-Padova	2020-10-01	Padua, Italy
NCT05175092	Living Donor Liver Transplantation for CRC Liver Metastases	/	2023-11	Wisconsin, United States
NCT03488953	Living Donor Liver Transplantation With Two Stage Hepatectomy for Patients With Isolated, Irresectable Colorectal Liver Metastases	LIVERT(W)OHEAL	2018-04-10	Jena &Tübingen, Germany
NCT06069960	SALT for Unresectable Colorectal Liver Metastases	SALT	2023-10-20	Shanghai, China

Table displaying clinicals trails, database last searched October 31 st, 2025. Any kind of trials on LT for patients with CRLM with the following recruitment statuses: “recruiting”, “active, not recruiting”, “not yet recruiting” or “enrolling by invitation” were taken into consideration

## Discussion

As patients with CRLM without local treatment options face a dismal prognosis, LT has regained considerable attention among surgeons and clinicians as a potentially curative, life-saving treatment modality, driven by growing evidence in this field. In this review, we provide an overview on the current scientific discussion regarding the topic and outline key requirements for translation of this treatment option from clinical trials to structured clinical programs and eventually clinical routine.

Based on the reviewed literature, LT should be considered an innovative rather than experimental treatment option for patients with liver-limited colorectal metastases that are neither resectable nor amendable to interventional therapies. Early data from the SECA-I trial had already demonstrated a 60% five-year survival rate for patients who previously had limited therapeutic options [25]. The TransMet trial confirmed these findings in a well-designed RCT, showing a five-year overall survival rate of approximately 73% for transplanted patients compared to 9% in those receiving chemotherapy alone [23]. The high number of currently active clinical trials is underlining the importance of this topic and suggesting that this intervention is becoming clinical routine in several countries soon. Moreover, comparing the outcome of transplanted patients in the TransMet trial vs. the 54.5% pooled 5-year overall survival found in a large multicenter study of patients transplanted with HCC, which is one of the bestestablished indications for liver transplantation so far and to date the cornerstone of transplant oncology, highlights the potential success of this treatment [29]. Although direct comparison of LT for CRLM and HCC are not feasible due to the different tumor biology, the outcome of LT for well-selected CRLM patients is remarkable.

Careful patient selection was identified as a key factor for success in published studies. Factors such as fully resected primary tumors, liver-only metastases, stable or responsive disease under chemotherapy, limited initial tumor burden and favorable tumor biology of the colorectal primary without BRAF V600E mutation or MSI-H/dMMR status [27] were found to be important variables in choosing the right patients for LT. Scoring systems such as the "Oslo score," integrating tumor size, CEA levels, chemotherapy response and disease duration could be a tool to effectively screen patient collectives for eligibility [27].

Although ‘tumor biology’ seems to be of great significance, especially regarding recurrence, it was found to be a loosely defined term characterized by surrogate variables in most studies identified by our search. This, paired with unclear existing interplays between tumor biology, chemotherapy response and waiting time are difficult to disentangle and warrant further investigation. Overall, two main strategies were identified to decrease the risk of recurrence: extending the waiting period under chemotherapy or integrating functional imaging technology to the evaluation process. In most ongoing studies, the waiting period under chemotherapy is consistently longer than 3 months, like in the above discussed University of Toronto clinical trial [30].

The PET-MTV has been described as an innovative approach that allows the quantitative summation of PET-avidity over all identifiable lesions. MTV provides a numeric representation of the overall disease burden throughout the liver. Grut et al. [27] were able to establish that a PET-MTV cutoff of ≥70 cm³ predicted both recurrence and survival. This approach was recently validated using a cohort of 26 patients undergoing LT for non-resectable CRLM at two academic transplant centers [31]. In this study, median follow-up was 609 days and time from PET to LT was 1.9 months, which is comparable to the findings from the TransMet trial. A PET-MTV ≥ 70 cm3 was associated with reduced RFS and overall survival after LT for CRLM, confirming findings from the Norway group. MTV measurements could therefore be a helpful tool for identification of suitable candidates for LT.

Despite overall promising survival rates, implementation of LT in clinical practice faces several challenges. Even carefully selected patients frequently experience recurrence, with nearly 75% of all participants in the SECA-I trial encountering recurrence within one-year post-transplant [25]. However, many of these recurrence events are surgically manageable. LT might therefore become part of the multimodal oncological algorithm to transfer nonresectable CRLM to a chronic disease rather than then being a curative intervention.

Ethical and practical issues surrounding organ scarcity should also be taken into consideration. Allocating donor livers from the already limited pool to metastatic cancer patients is historically controversial, given competing demands from patients with end-stage liver disease, other well-established indications such as HCC, or relatively new indications such as alcoholic liver disease. This is especially true in countries with low donation rates such as Germany with a rate of only 10.4 organs donation per million inhabitants in 2024 [32]. Unter these conditions, the utilization of severely marginal grafts that have qualified after viability assessment by normothermic machine perfusion could be a viable solution – although this concept has not been studied in CRLM patients so far [33]. LDLT offers an alternative to the deceased donor pool but introduces ethical concerns regarding donor safety and justification as discussed by Byrne et al. [22, 34]. Moreover, LDLT is ethically challenging when donors should assume risk for recipients with high recurrence. Another option could be using organs from donors with a history of cancer, as currently investigated by the TransMIT trial [35]. While both options offer a possible route to organ transplantation to the individual patient, the inherent risk of both options for either donor or recipient must be considered and appropriately addressed during consultation with the individual patient. Balancing innovation and ethical responsibilities remain a critical ongoing challenge. This overall optimistic jet cautious stance is echoed by Varely et al. in their 2021 systematic review and Meta-Analysis. They also stressed the importance of larger patient populations in upcoming trials [36].

Additionally, policy adjustments are necessary to ensure fast but also fair access for CRLM patients without disadvantaging others. In Germany, this could be accomplished by establishing Standard Exceptions for patients with unresectable CRLM to account for the mortality risk not reflected by the scoring systems used for allocation, i.e. the refitted MELD-Natrium score. In addition, the implementation of efficient, independent institutions such as national tumor boards to review and approve listing of CRLM patients for transplantation is necessary, as successfully piloted in the TransMet trial.

In line with ethical considerations, quality of life represents a critical outcome. Patients undergoing LT are exposed to lifelong immunosuppression, with associated metabolic, infectious, and oncological risks, as well as the psychological and logistical burden of intensive post-transplant surveillance. In line with IHPBA recommendations [37], future trials as well as programs should therefore incorporate standardized QoL assessments to better contextualize survival benefits against long-term treatment burden and to support informed patient selection and shared decision-making.

Our narrative review has several limitations, particularly regarding the grade of evidence and the risk of bias, with robust data from only one RCT available so far. While some of the studies included in our review were less methodologically robust, the authors still found them to be convincing.

On this basis, a non-randomized, non-blinded multicenter study is currently under preparation in Germany, which is supported by all transplant centers and the Working Group on Internal Oncology in the German Cancer Society (German Transplant Oncology Trial [GTO] 001, AIO-KRK-0325). This study is designed to establish a structured LT program for highly selected patients with CRLM without local treatment options in Germany and will generate real-world data on this innovative treatment approach. While the overall concept, i.e. LT in highly selected patient with CRLM without local treatment options, appears to be justified by the randomized TransMet trial, questions such as of prognostic factors or the benefit of LT if borderline-resectable patients should be addressed in future prospective studies. Moreover, quality of life under immunosuppression needs to be included in future trials consideration, and treatment of recurrence must be strictly monitored.

## Conclusion

In conclusion, LT for unresectable CRLM is reaching a stage where a clinical implementation in structured programs must be seriously considered. However, this intervention can only succeed if all aspects of transplantation medicine, together with strict selection criteria, are considered. Precise preoperative risk stratification based on chemotherapeutic response, molecular markers, and functional imaging is critical to treatment success. Additionally, prioritization on the waiting list, e.g. by implementation of standard exception MELD scores for this disease, would be necessary to provide this treatment option at scale. If these prerequisites are met, LT may develop from an innovative approach to a routine element of the already highly individualized and interdisciplinary care of patients with metastatic colorectal cancer.

Flowchart displaying identification, screening, eligibility assessment, and inclusion of studies in our narrative review. The original database search resulted in 1574 records from MEDLINE. After title and abstract screening of these unique records, 57 records were identified for retrieval. Of these, 11 reports could not be retrieved. Forty-six full-text reports were assessed for eligibility; 40 were excluded for the following reasons: 26 did not fit the cope of the review, 13 represented repetitive data reporting, and one had a sample size below the study threshold auf five cases. Six studies were included in the final review.

Cochrane Risk of Bias (RoB 2.0) assessment. Domain definitions are: D1 – bias arising from the randomization process; D2 – bias due to deviations from intended intervention; D3 – bias due to missing outcome data; D4 – bias in measurement of the outcome; and D5 – bias in selection of the reported result). Domain-level judgments are represented by colored “traffic-light” symbols: green circles with a plus sign indicate low risk of bias, yellow circles indicate moderate risk, and red circles with an “X” indicate serious risk of bias for that domain. All five domains were judged as low risk of bias, and the overall risk of bias for this study was also determined to be low.

Risk-of-bias assessment of five included studies using the ROBINS-I tool. Each study (Dueland et al. [25], Grut et al. [23], Sasaki et al. [26], Kaltenmeier et al. [24], and Byrne et al. [22]) was evaluated across seven bias domains defined by ROBINS-I listed above. Domain-level judgments are represented by colored “traffic-light” symbols: green circles with a plus sign indicate low risk of bias, yellow circles indicate moderate risk, and red circles with an “X” indicate serious risk of bias for that domain. The column on the right displays the overall risk-of-bias rating for each study, corresponding to the highest level of bias observed across its domains. Of the five studies, three (Dueland et al. [25], Sasaki et al. [26], and Kaltenmeier et al. [24]) exhibit a serious risk of bias due to confounding (D1), as indicated by red symbols in the D1 column, which drives their overall risk-of-bias to be judged as serious (red in the Overall column). The remaining two studies (Grut et al. [23] and Byrne et al. [22]) show low risk of bias (green) in most domains, with only moderate concerns (yellow) in one or two areas (for example, a moderate risk of bias due to confounding in D1), resulting in overall moderate risk-of-bias judgments for these studies. Confounding (D1) was the primary source of bias in these non-randomized studies, whereas biases in other domains (D3–D6) were generally rated as low risk for most studies.

Geographic representation of currently active clinical trials on LT for CRLM listed in ClinicalTrials.gov. In total, 21 clinical trials were identified. Of those, 5 were conducted in the Nordic countries, 5 in Italy, 3 in the United States of America, 2 in Spain and China and one in Germany, South Korea and Canada, respectively.

## Data Availability

No datasets were generated or analysed during the current study.

## Abbreviations

18F: FDG—fluorine—18 fluorodeoxyglucose
5: FU—5—fluorouracil
BRAF V600E: BRAF V600E mutation
CEA: carcinoembryonic antigen
CI: confidence interval
CRC: colorectal cancer
CRLM: colorectal liver metastases
DDLT: deceased—donor liver transplantation
dMMR: deficient mismatch repair
ECOG: Eastern Cooperative Oncology Group (performance status)
EGFR: epidermal growth factor receptor
ESMO: European Society for Medical Oncology
FOLFOXIRI: 5—FU/leucovorin + oxaliplatin + irinotecan
FOLFIRI: 5—FU/leucovorin + irinotecan
HAI: hepatic artery infusion (chemotherapy)
HCC: hepatocellular carcinoma
HIV: human immunodeficiency virus
HR: hazard ratio
LDLT: living—donor liver transplantation
LT: liver transplantation
MELD: Model for End—Stage Liver Disease
MELD: Na / MELD—Natrium—MELD sodium score
MSI: H—microsatellite instability—high
MTV: metabolic tumor volume
OS: overall survival
PET: positron emission tomography
PET/CT: positron emission tomography / computed tomography
PRISMA: P—Preferred Reporting Items for Systematic review and Meta—Analysis Protocols
PubMed: PubMed database
R0: margin—negative (microscopically complete) resection
RCT: randomized controlled trial
RECIST: Response Evaluation Criteria in Solid Tumors
RFA: radiofrequency ablation
RFS: recurrence—free survival
RoB 2: revised Cochrane Risk—of—Bias tool for randomized trials
ROBINS: I—Risk Of Bias In Non—randomized Studies of Interventions
SBRT: stereotactic body radiotherapy
SECA/SECA: I—SECA study/study phase (as written)
SM: author initials (as written)
TACE: transarterial chemoembolization
TRANSMET/TransMet/TransMET: trial name (as written)
TransMIT: trial name (as written)
VEGF: vascular endothelial growth factor
